# Characteristics and clinical significance of lipid metabolism in patients with gastrointestinal stromal tumor

**DOI:** 10.1186/s12944-021-01613-7

**Published:** 2022-01-07

**Authors:** Xiaoling Liu, Jun Hu, Bende Liu

**Affiliations:** 1 Department of Endocrinology, Liyuan Hospital, Tongji Medical College, Huazhong University of Since and Technology, 39Yanhu Avenue, Hongshan District, Wuhan, 430022 Hubei China; 2 Department of Union Jiangnan Hospital Huazhong University of Science and Technology, 1 Wenhua Avenue, Jiangxia District, Wuhan, 430022 Hubei China; 3 Department of Cardiovascular system Gastrointestinal Surgery, Union Hospital, Tongji Medical College, Huazhong University of Science and Technology, 1277 Jiefang Avenue, Jianghan District, Wuhan, 430022 Hubei China

**Keywords:** Gastrointestinal stromal tumor, Lipid metabolism, Clinical pathology, GIST, Lipid, Risk stratification

## Abstract

**Background:**

To investigate the characteristics and clinical significance of serum lipids in patients with gastrointestinal stromal tumors (GISTs).

**Methods:**

The clinical and pathological data of 694 GIST patients in Liyuan hospital and Union hospital from 2012 to 2016 were retrospectively analyzed. Blood lipid levels in patients with varying degrees of risk were compared.

**Results:**

The findings showed that LDL-C, HDL-C, and CHOL increased significantly in women, and CD34 positive. In patients with tumors size less than 5 cm in diameter, TG, HDL-C, and CHOL were significantly higher. TG levels were significantly higher in DOG-1 (a marker and has a high specificity and sensitivity in the diagnosis of GIST) positive patients than in DOG-1 negative patients (*P* < 0.05). S-100 positive patients had lower HDL-C levels than S-100 negative patients (*P* < 0.05). Lipids indexes were found to be correlated with GIST risk stratification and tumor site (*P* < 0.05). TG/HDL-C was were significantly different among patients with GIST in different locations (*P* < 0.05).

**Conclusion:**

The clinical and pathological characteristics of the patients with GIST are closely related to the level of blood lipids. To a certain extent, information about level of blood lipids can be helpful for distinguishing benign and malignant GIST.

## Background

Gastrointestinal stromal tumors (GISTs) originate from Cajal cells. GIST is the most common tumor originating from gastrointestinal mesenchymal tissue, with an incidence of approximately 0.128 per billion population in China [1, 2]. GIST, which can manifest itself anywhere through gastrointestinal tract, has the biggest incidence rate in stomach, followed by the duodenum. Lipid metabolism disorder has been related with a large of malignant tumors, such as thyroid cancer, lung cancer, liver cancer, stomach cancer, colorectal cancer, and so on [3]. Gastrointestinal stromal tumors can be classified into low, medium and high malignant levels according to their clinicopathological characteristics [4]. At present, it is difficult to differentiate benign from malignant gastrointestinal stromal tumors, and EUS-FNA (endoscopic ultrasonography-guided fine needle aspiration) is the most accurate, reliable, receptive, and safe testing method in clinical practice [5]. Only a few studies have been conducted to date on abnormal lipid metabolism in GIST patients and its association to the degree of malignancy. For the first time, 694 patients with gastrointestinal stromal tumors had their preoperative lipid levels retrospectively analyzed, to further elucidate the relationship between lipid levels and the clinicopathological characteristics of patients, which aids in the differentiation of benign from malignant GISTs.

## Method

### Patients

Seven hundred forty-one patients with gastrointestinal stromal tumors who underwent surgical sugery from 2012 to 2016 at Union Hospital and Liyuan Hospital of Huazhong University of Since and Technology were selected. All patients were diagnosed with gastrointestinal stromal tumors in accordance with the ESMO Clinical Practice Guidelines (2012) [6]. It is the accepted guideline for diagnosis and treatment of GIST. Risk stratification was performed using NIH 2008 modified classification and AFIP classification. The inclusion criteria were as follows: (1) clear pathological and immunohistochemical diagnosis; (2) complete clinical data; (3) no history of serious diabetes; and (4) no history of serious cardiopulmonary disease. The exclusion criteria were following: (1) absence of pathological data; (2) presence of other malignant tumors; (3) history of severe diabetes; (4) history of the severe cardiopulmonary disease; and (5) use of statins, fibrates or any other lipid-lowering medication. The risk of developing cancer was classified according to tumor size and its mitotic index [7]. The trial was conducted in accordance with the principles of the Declaration of Helsinki. It was approved by approved by the hospital ethics board. All information was accessed after receiving informed consent from the patients.

### Specimen collection and detection

Each patient had 5 ml fasting venous blood drawn 1–2 days following admission to the hospital, and serum was prepared within 2 h. Triglyceride (TG), low density lipoprotein cholesterol (LDL-C), high density lipoprotein - cholesterol (HDL-C), total cholesterol (CHOL), and TG/ HDL-C were detected on serum specimens. TG was determined by the GPO-PAP method, HDL-C, and LDL-C by the direct method, and CHOL by the CHOD-PAP method. The Department of Clinical Laboratory, Union Hospital of Huazhong University of Science and Technology established the reference ranges for normal values: TG 0.050 ~ 1.700 mmol/L, LDL-C < 3.370 mmol/L, HDL-C 1.040 ~ 1.550 mmol/L, and CHOL 2.900 ~ 5.200 mmol/L.

### Statistical method

Statistical analyses of the data were conducted on SPSS 23 software. The relationship between numerous factors and blood lipids was analyzed by one-way analysis of variance. *P*<0.05 was defined as significant statistical difference. The counting data were represented as (X ± s), while the measurement data were represented by the chi-square value. For data with more than two measurements, a single factor anova wasused, and Graphpad was used to calculate the box graph illustrating the relationship between different risk stratifications and lipid levels.

## Results

### Clinical data of all included cases

**Fig. 1:**
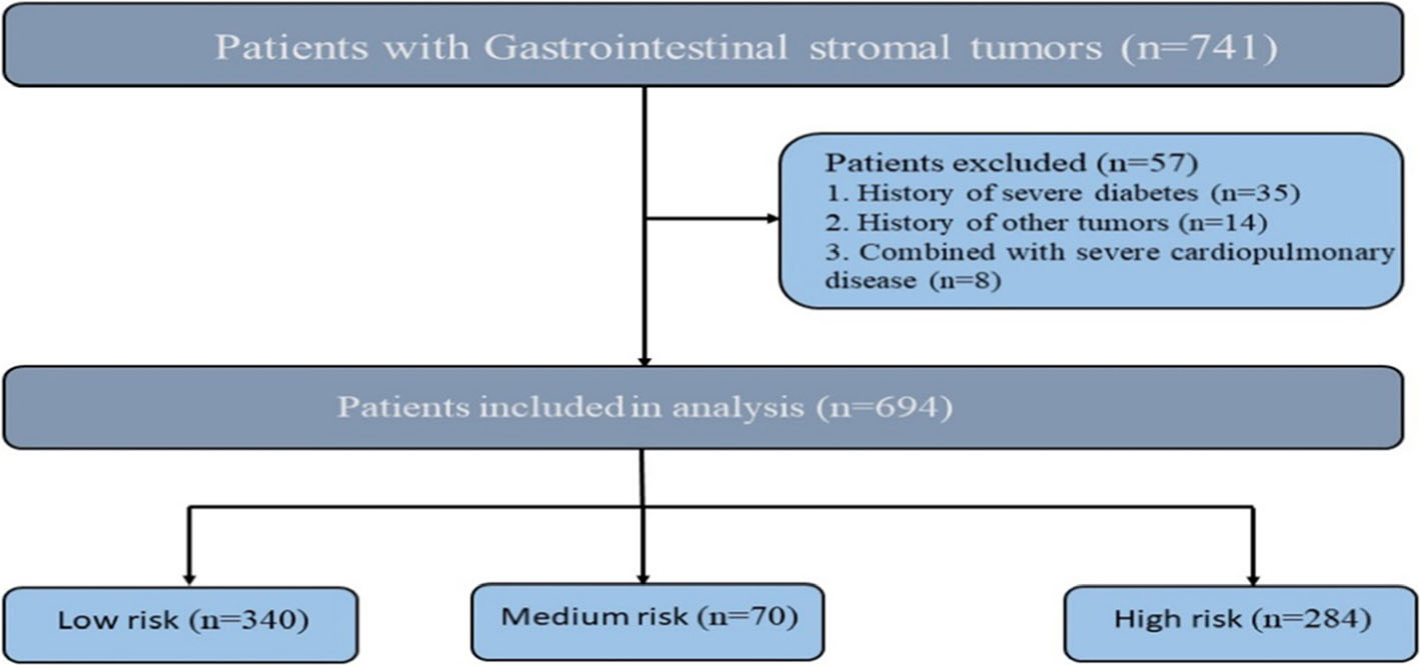
Flowchart of the patients with gastrointestinal stromal tumors selection. 694GISTs are included in this analysis, including 340patients in low risk, 70patients in medium risk, 284patients in high risk. Fifty-six were using statins and other lipid-lowering drugs

Of the 741 patients with GISTs recruited in this study, 56 were using statins or other lipid-lowering drugs. According to the inclusion and exclusion criteria, a total of 694 patients with gastrointestinal stromal tumors were eligible, including 388 males and 306 females. According to the NIH 2008 modified version and AFIP classification, there were 340 cases classified as low-risk, 70 cases medium-risk, and 284 cases high-risk (Fig. 1). The mean age was 57.63 ± 10.94 years. Five hundred eighty-one patients were diagnosed with a single tumor, while 113 patients had multiple tumors. Tumors in the esophagus, stomach, duodenum, hollow ileum, colorectal and gastrointestinal tract occurred at a rate of 1.44, 55.19, 8.36, 18.59, 4.03 and 12.39%, respectively. The proportion of tumor tissue with a diameter less than 5 cm and greater or equal to 5 cm was 55.33 and 44.67%, respectively.

### Relationship between clinical characteristics and various blood lipid indexes

The age of the patient and the presence of single or multiple tumors had no discernible effect on lipid levels. Female patients had higher levels of LDL-C, HDL-C, and CHOL levels than male patients. TG and TC levels in patients with tumor size ≥5 cm were higher than in tumor size < 5 cm. There were significant differences between serum lipids and TG/ HDL-C levels at distinct mesenchymal sites (all *P* < 0.05) (Table 1).

**Table 1:** the relation between clinical significance and blood lipids in GISTs(X ± s)

	n	TG	P	LDL-C	P	HDL-C	P	CHOL	P	TG/HDL-C	P
		(mmol/L)		(mmol/L)		(mmol/L)		(mmol/L)			
Sex											
Male	388	1.28 ± 0.79	0.223	2.39 ± 0.73	0.000	1.08 ± 0.30	0.000	3.97 ± 0.89	0.000	1.33 ± 1.06	0.526
Female	306	1.37 ± 1.13		2.61 ± 0.82		1.27 ± 0.36		4.44 ± 1.06		1.27 ± 1.55	
Age											
< 60y	312	1.27 ± 0.79	0.188	2.49 ± 0.79	0.928	1.17 ± 0.35	0.920	4.17 ± 1.01	0.848	1.25 ± 1.07	0.276
≥ 60y	382	1.36 ± 1.07		2.48 ± 0.77		1.16 ± 0.33		4.18 ± 0.99		1.35 ± 1.46	
Tumor cites											
Esophageal	10	1.24 ± 0.79	0.08	2.19 ± 0.53	0.000	1.18 ± 0.49	0.000	3.75 ± 0.79	0.000	1.29 ± 0.97	0.002
Stomach	383	1.35 ± 0.99		2.60 ± 0.78		1.22 ± 0.33		4.35 ± 0.99		1.24 ± 1.14	
Duodenum	58	1.53 ± 1.18		2.35 ± 0.82		1.07 ± 0.37		4.02 ± 0.99		1.79 ± 1.98	
Jejunoileum	129	1.33 ± 1.05		2.21 ± 0.72		1.03 ± 0.33		3.79 ± 0.94		1.51 ± 1.70	
Colorectal	28	0.99 ± 0.34		2.67 ± 0.97		1.28 ± 0.27		4.38 ± 1.03		0.84 ± 0.49	
External gastrointestinal tract	86	1.14 ± 0.46		2.48 ± 0.78		1.15 ± 0.34		4.06 ± 0.94		1.10 ± 0.59	
Tumor size											
< 5 cm	384	1.42 ± 1.10	0.004	2.54 ± 0.80	0.060	1.21 ± 0.35	0.000	4.29 ± 1.00	0.000	1.36 ± 1.39	0.230
≥ 5 cm	310	1.21 ± 0.72		2.43 ± 0.75		1.11 ± 0.33		4.03 ± 0.98		1.24 ± 1.16	
Multiple or not											
Yes	113	1.29 ± 0.57	0.736	2.51 ± 0.68	0.770	1.14 ± 0.31	0.475	4.19 ± 0.87	0.896	1.24 ± 0.71	0.557
Not	581	1.33 ± 1.01		2.49 ± 0.79		1.17 ± 0.35		4.17 ± 1.02		1.32 ± 1.38	

### Relationship between tumor pathology and various lipid indexes

Tumor markers such as CD117, SMA, and Ki67 had no effect on preoperative serum lipid levels. Besides LDL-C, HDL-C and CHOL levels were higher in CD34 positive patients than in CD34 negative patients. TG levels were significantly greater in the DOG-1 positive patients than in DOG-1 negative ones (*P* < 0.05). S-100 positive patients had lower HDL-C levels than S-100 negative patients. TG/ HDL-C ratio was lower in CD34 or DOG-1 positive, compared to CD34 or DOG-1 negative patients (all *P* < 0.05) (Table 2).

**Table 2:** the relation between pathological significance and blood lipids in GISTs(X ± s)

	n	TG	P	LDL-C	P	HDL-C	P	CHOL	P	TG/HDL-C	P
		(mmol/L)		(mmol/L)		(mmol/L)		(mmol/L)			
CD117											
Positive	665	1.32 ± 0.96	0.860	2.49 ± 0.78	0.810	1.16 ± 0.34	0.336	4.18 ± 0.99	0.804	1.31 ± 1.31	0.991
Negative	9	1.27 ± 0.46		2.43 ± 0.59		1.05 ± 0.35		4.09 ± 0.92		1.31 ± 0.77	
CD34											
Positive	574	1.31 ± 0.91	0.555	2.54 ± 0.78	0.000	1.19 ± 0.34	0.000	4.24 ± 0.99	0.000	1.26 ± 1.14	0.010
Negative	98	1.38 ± 1.19		2.24 ± 0.70		1.02 ± 0.33		3.83 ± 0.96		1.62 ± 1.99	
DOG-1											
Positive	663	1.31 ± 0.85	0.000	2.49 ± 0.78	0.758	1.17 ± 0.34	0.125	4.18 ± 0.99	0.985	1.29 ± 1.22	0.001
Negative	9	2.47 ± 3.94		2.42 ± 0.69		0.99 ± 0.34		4.17 ± 0.91		2.77 ± 4.31	
SMA											
Positive	231	1.41 ± 1.24	0.089	2.44 ± 0.79	0.173	1.13 ± 0.34	0.147	4.11 ± 1.02	0.198	1.42 ± 1.65	0.127
Negative	441	1.28 ± 0.76		2.52 ± 0.77		1.17 ± 0.34		4.21 ± 0.99		1.26 ± 1.09	
S-100											
Positive	16	1.32 ± 0.71	0.978	2.46 ± 0.93	0.862	0.98 ± 0.33	0.031	3.96 ± 1.18	0.372	1.66 ± 1.34	0.28
Negative	657	1.32 ± 0.96		2.49 ± 0.77		1.17 ± 0.34		4.18 ± 0.99		1.30 ± 1.31	
Ki67%											
< 5%	395	1.34 ± 1.08	0.583	2.47 ± 0.74	0.409	1.18 ± 0.34	0.081	4.18 ± 0.92	0.886	1.32 ± 1.46	0.875
≥ 5%	269	1.30 ± 0.75		2.53 ± 0.82		1.13 ± 0.34		4.17 ± 1.09		1.30 ± 1.06	
Rank of risk^a^											
Extremely low risk	117	1.34 ± 0.83	0.012	2.62 ± 0.80	0.033	1.27 ± 0.36	0.000	4.44 ± 0.95	0.000	1.24 ± 1.21	0.169
Low risk	224	1.48 ± 1.27		2.47 ± 0.75		1.17 ± 0.34		4.19 ± 0.97		1.46 ± 1.56	
Medium risk	70	1.28 ± 0.60		2.64 ± 0.80		1.20 ± 0.32		4.38 ± 1.07		1.17 ± 0.69	
High risk	283	1.20 ± 0.75		2.41 ± 0.79		1.11 ± 0.33		4.01 ± 0.99		1.24 ± 1.21	

^a^ Extremely low risk was defined as a GIST with tumor size ≤2 cm and arbitrary mitotic index, or 2 cm < tumor size ≤5 cm and mitotic index < 5 per 50 HPF. low risk was defined as a GIST with 5 cm < tumor size ≤10 cm and mitotic index < 5 per 50 HPF. Medium risk was defined as a GIST with tumor size > 10 cm and mitotic index < 5 per 50 HPF, or 2 cm < tumor size ≤5 cm and mitotic index ≥5 per 50 HPF. High risk was defined as a GIST with tumor size > 5 cm and mitotic index ≥5 per 50 HPF

### Relationship between risk groups and various blood lipid indexes

**Fig. 2:**
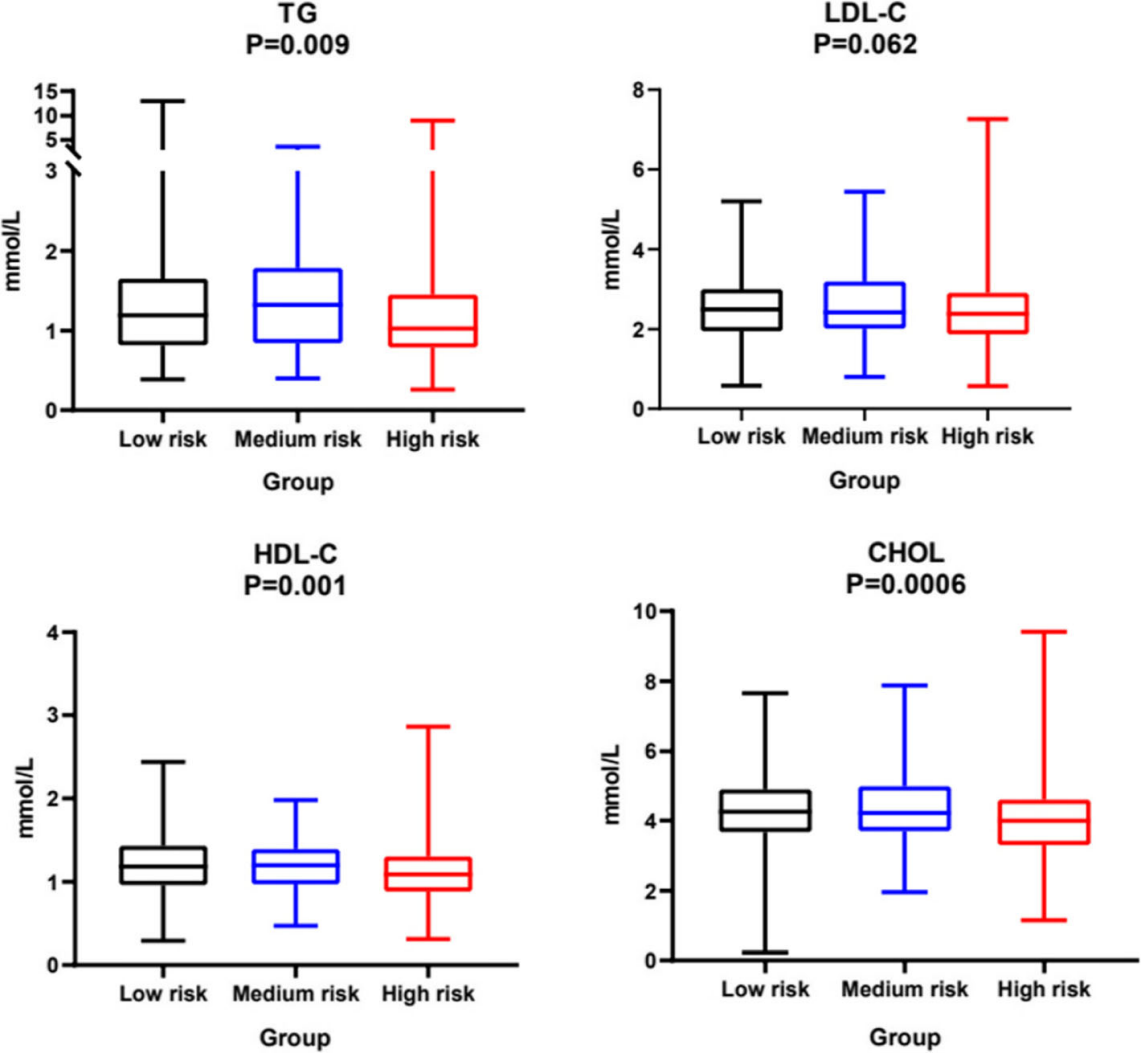
Differences in patients with different risk grades Box plot of the lipids level in various risk groups. There are significant difference in TG、HDL-C, and CHOL with risk sub-groups

Blood lipid levels were significantly different in GIST patients with varying degrees of malignancy (all *P* < 0.05) (size effect: η^2^ = 0.21). Compared to low-risk patients, high-risk patients had lower lipids levels, including TG, HDL-C, LDL-C, and CHOL. While TG/ HDL-C values did not exist any difference in patients with different risk levels (Fig. 2).

## Discussion

The blood lipid levels of 694 patients with gastrointestinal stromal tumor were analyzed in this study. The findings suggested that multiple factors, including gender, tumor site, tumor size, CD34, CD117, and S-100 were related to blood lipid levels in patients with gastrointestinal stromal tumors. S-100 protein is a kind of the calcium-binding proteins, presenting in most gastrointestinal tumors. However, GIST is mostly negative for S-100. Patients with highly malignant gastrointestinal stromal tumors had lower lipids levels than in patients with other risk stratifications of stromal tumors. We hypothesized that GIST patients had a lipid metabolism disorder, which could have an influence on the degree of malignancy of the tumor.

Numerous studies have linked gastrointestinal stromal tumors to gene mutations in tyrosine kinase receptor (KIT) and platelet-derived growth factor receptor-alpha (PDGFRA). However, wild-type KIT /PDGFRA can be discovered in about 10–15% GISTs [8]. Clinicopathological characteristics of patients with GIST are complex and are influenced by several factors. KIT/PDGFRA may exert an effect on lipid metabolism via distinct signal transduction pathways [9]. Most tumor patients, such as those with thyroid cancer, lung cancer, or liver cancer, have lipid metabolism disturbance, and elevated lipid levels have been shown to promote the growth of multiple tumor types [10–12]. Obesity, on the other hand, has been demonstrated in some studies to play a protective effect on gastrointestinal stromal tumors [13]. In the 1920s, Warburg demonstrated, for the first time that tumor cells prioritize anaerobic fermentation even when oxygen supply is adequate, providing tumor cells with sufficient energy and prerequisite material for the synthesis of biological macromolecules, a phenomenon termed as the Warburg effect [14]. Warburg effect indicates that tumor cells require energy not only for growth but also for the synthesis of a vast number of macromolecules. Aerobic glycolysis not only increases tumor cells’ energy production efficiency, but also provides necessary conditions for the synthesis of biomolecular components necessary for growth, adhesion, metastasis, and other biological characteristics [15, 16]. Given the critical roles that lipids play in cellular membrane formation, energy and macromolecular metabolism, cellular signal transduction and other cellular activities, it is not surprising to find that associations between lipid metabolism and tumorigenesis have been discovered. In this study, by examining data from 694 GIST patients, we demonstrated that lipid metabolism was associated with tumor size, tumor site, tumor molecular markers, and the risk level in GIST patients.

There is more and more evidence that lipid metabolism has a vital role in tumor progression. The lipid raft, which is rich in neurolipids and cholesterol, is a unique small lipid domain on the cell membrane that acts as a selective signal transducer, regulating lipid metabolism, tumor cell growth, adhesion, and metastasis, and promoting tumor progression [17]. On the other hand, provide energy to tumor cells as well. To meet the needs of tumor cells’ rapid growth, lipid metabolism is expedited and lipid content is reduced. Fatty acid synthase overexpression may significantly contribute to the elevation of lipid levels in GIST patients [18]. In terms of nutritional support, abnormal host metabolism might result in the mobilization of host tissues and inefficient supplementation in patients with malignant tumors. Increased fat breakdown, decreased or increased fat production, increased free fatty acids (FFA) and glycerol turnover, and increased FFA-triacylglycerol circulation all contribute to lipid metabolism dysfunction [19]. Additionally, lipids are required for the synthesis of signaling molecules that can alter the microenvironment of tumor cells and promote tumor cell growth [20]. Additionally, HDL plays a critical role in maintaining proper intracellular cholesterol homeostasis by binding to cell membrane surface receptors and removing excess cholesterol from the cell [21].

Recent evidence suggests that TG/ HDL-C can be utilized as an independent risk factor for predicting the prognosis of breast cancer and gastric tumor and that its predictive value is superior to that of TG [22]. The ratio of TG/ HDL-C may be related to insulin resistance, which is associated with gastrointestinal tumors such as gastric cancer. The results of this study indicated that the ratio of TG/ HDL-C was different in gastrointestinal stromal tumors with varying clinical characteristics, implying that insulin resistance may be associated with gastrointestinal stromal tumors [23].

Compared with other studies, this study focuses on systematic analysis of laboratory examination data from a clinical perspective. As the number of cases included was large enough in this study, the results were more reliable in comparison to some other studies. The results of this study may have guiding significance for GIST patients’ dietary selection, suggesting that patients adhere to low-fat diet. In the future, the molecular mechanism of abnormal lipid metabolism in GIST patients may be further explored.

## Strengths and limitations

This study innovatively analyzed the dyslipidemia of patients with GISTs. The limitation of this study is in that it failed to compare the changes in lipid levels in GIST patients before and after surgery. In addition, the effect of serum lipid levels on the prognosis of patients with gastrointestinal stromal tumors is still unclear, and further studies are needed to confirm this in the future. Whether prior use of lipid-lowering drugs such as statins or betas had any effect on the study is unknown.

## Conclusion

In general, patients with GISTs have varying degrees of lipid metabolism disorders. It also offers a new perspective for exploring abnormalities in GIST metabolism in this study. Therefore, both treatment and nursing on them should be taken seriously.

## Data Availability

Not applicable.

## References

[1] Blay JY, Bonvalot S, Casali P, Choi H, Debiec-Richter M, Dei Tos AP, Emile JF, Gronchi A, Hogendoorn PCW, Joensuu H (2005). Consensus meeting for the management of gastrointestinal stromal tumors report of the GIST consensus conference of 20–21 March 2004, under the auspices of ESMO. Ann Oncol.

[2] Corless CL, Barnett CM, Heinrich MC (2011). Gastrointestinal stromal tumours: origin and molecular oncology. Nat Rev Cancer.

[3] Lee GB, Lee JC, Moon MH (2019). Plasma lipid profile comparison of five different cancers by nanoflow ultrahigh performance liquid chromatography-tandem mass spectrometry. Anal Chim Acta.

[4] Joensuu H (2008). Risk stratification of patients diagnosed with gastrointestinal stromal tumor. Hum Pathol.

[5] Akahoshi K, Oya M, Koga T, Shiratsuchi Y (2018). Current clinical management of gastrointestinal stromal tumor. World J Gastroenterol.

[6] Casali PG, Abecassis N, Aro HT, Bauer S, Biagini R, Bielack S, Bonvalot S, Boukovinas I, Bovee JVMG, Brodowicz T, et al. Gastrointestinal stromal tumors: ESMO clinical practice guidelines for diagnosis, treatment and follow-up. Ann Oncol. 2012;23:i49–i55. 10.1093/annonc/mdy095. 10.1093/annonc/mds252 22997454

[7] Steigen SE, Eide TJ (2009). Gastrointestinal stromal tumors (GISTs): a review. APMIS.

[8] Heinrich MC, Corless CL, Duensing A, McGreevey L, Chen C, Joseph N, Singer S, Griffith DJ, Haley A, Town A (2003). PDGFRA activating mutations in gastrointestinal stromal tumors. Science.

[9] Li C, Fang F, Chen Y, Liu T, Chan T, Yu S, Chen L, Huang H (2017). Overexpressed fatty acid synthase in gastrointestinal stromal tumors: targeting a progression-associated metabolic driver enhances the antitumor effect of imatinib. Clin Cancer Res.

[10] Liu T, Peng F, Yu J, Tan Z, Rao T, Chen Y, Wang Y, Liu Z, Zhou H, Peng J (2019). LC-MS-based lipid profile in colorectal cancer patients: TAGs are the main disturbed lipid markers of colorectal cancer progression. Anal Bioanal Chem.

[11] Cristea S, Coles GL, Hornburg D, Gershkovitz M, Arand J, Cao S, Sen T, Williamson SC, Kim JW, Drainas AP (2020). The MEK5–ERK5 kinase axis controls lipid metabolism in small-cell lung cancer. Cancer Res.

[12] Hu B, Lin JZ, Yang XB, Sang XT (2020). Aberrant lipid metabolism in hepatocellular carcinoma cells as well as immune microenvironment: a review. Cell Prolif.

[13] Stiles ZE, Rist TM, Dickson PV, Glazer ES, Fleming MD, Shibata D, Deneve JL (2017). Impact of body mass index on the short-term outcomes of resected gastrointestinal stromal tumors. J Surg Res.

[14] Liberti MV, Locasale JW (2016). The Warburg effect: how does it benefit cancer cells?. Trends Biochem Sci.

[15] Skotland T, Kavaliauskiene S, Sandvig K (2020). The role of lipid species in membranes and cancer-related changes. Cancer Metastasis Rev.

[16] Pakiet A, Kobiela J, Stepnowski P, Sledzinski T, Mika A (2019). Changes in lipids composition and metabolism in colorectal cancer: a review. Lipids Health Dis.

[17] Luo X, Cheng C, Tan Z, Li N, Tang M, Yang L, Cao Y (2017). Emerging roles of lipid metabolism in cancer metastasis. Mol Cancer.

[18] Li C, Liu T, Chuang I, Chen Y, Fang F, Chan T, Li W, Huang H (2017). PLCB4 copy gain and PLCß4 overexpression in primary gastrointestinal stromal tumors: integrative characterization of a lipid-catabolizing enzyme associated with worse disease-free survival. Oncotarget.

[19] Körber J, Pricelius S, Heidrich M, Müller MJ (1999). Increased lipid utilization in weight losing and weight stable cancer patients with normal body weight. Eur J Clin Nutr.

[20] Snaebjornsson MT, Janaki-Raman S, Schulze A (2020). Greasing the wheels of the cancer machine: the role of lipid metabolism in cancer. Cell Metab.

[21] Dessì S, Batetta B, Pulisci D, Spano O, Anchisi C, Tessitore L, Costelli P, Baccino FM, Aroasio E, Pani P (1994). Cholesterol content in tumor tissues is inversely associated with high-density lipoprotein cholesterol in serum in patients with gastrointestinal cancer. Cancer Am Cancer Soc.

[22] Sun H, Huang X, Wang Z, Zhang G, Mei Y, Wang Y, Nie Z, Wang S (2019). Triglyceride-to-high density lipoprotein cholesterol ratio predicts clinical outcomes in patients with gastric cancer. J Cancer.

[23] Young KA, Maturu A, Lorenzo C, Langefeld CD, Wagenknecht LE, Chen YI, Taylor KD, Rotter JI, Norris JM, Rasouli N (2019). The triglyceride to high-density lipoprotein cholesterol (TG/HDL-C) ratio as a predictor of insulin resistance, β-cell function, and diabetes in Hispanics and African Americans. J Diabetes Complicat.

